# The neuromuscular junction: a critical component of functional recovery after peripheral nerve injury

**DOI:** 10.3389/fsurg.2026.1746207

**Published:** 2026-02-24

**Authors:** Whitney E. Muhlestein, Mark A. Mahan

**Affiliations:** Department of Neurosurgery, Clinical Neurosciences Center, University of Utah, Salt Lake City, UT, United States

**Keywords:** neuromuscular junction, peripheral nerve injury, peripheral nerve surgery, regeneration, translational science

## Abstract

Peripheral nerve injury can result in potentially devastating neurological deficits and often disproportionately impacts young workers. Recovery of motor function after peripheral nerve injury requires the regeneration of the nerve from the site of injury to the neuromuscular junction (NMJ), where signals must be transduced effectively across the synapse, resulting in target muscle contraction. For reasons that are not fully understood, after about 18 months of denervation, functional NMJs fail to recapitulate. This, in combination with the slow velocity of axon regeneration, significantly limits both the window of opportunity for intervention and surgical reconstruction options. Here, we review what is currently known with respect to NMJ physiology, anatomy, development, and changes after injury. We also highlight knowledge gaps and opportunities for study with the goal of developing novel, NMJ-focused avenues of treatment for patients after peripheral nerve injury.

## 1 Introduction

There are approximately 2,000 brachial plexus injuries and 17,000 injuries to major peripheral nerves annually in the United States (1, 2). These conditions disproportionately affect young workers and can result in significant lifelong pain, disability, and socioeconomic disadvantages. After nerve injury, Wallerian degeneration results in the disintegration of axons distal to the injury. For a recovering nerve to reform functional connections to its target muscle, it must regenerate along the entire distance from the point of injury to the neuromuscular junction (NMJ), a specialized synapse through which electrical signals originating in the central nervous system are transduced to skeletal muscle via motor neurons. The NMJ is composed of a presynaptic motor neuron terminus, a postsynaptic motor endplate characterized by a high concentration of acetylcholine receptors (AChRs), and a synaptic cleft—which is enriched with specialized extracellular matrix (ECM) components—separating the two. Increasingly recognized as crucial to the NMJ are terminal Schwann cells (tSCs), non-myelinating glial cells associated with motor nerve endings (3).

For reasons that are not fully understood, human NMJs that have been denervated for longer than approximately 18 months fail to regain functionality (4). Thus, for motor recovery to occur, the regenerating axon must reach the NMJ within a year and a half. However, because nerves regenerate at a rate of only 1 mm/day, some muscles may be too far away from the point of injury for successful innervation (5). Despite it being an essential gateway of regeneration, we still lack critical details in our understanding of the NMJ and its relationship to axonal loss.

## 2 Physiology

During skeletal muscle contraction, an action potential is propagated down the motor neuron, triggering the opening of voltage-gated calcium channels in the presynaptic motor neuron terminus. Calcium ions flow into the motor neuron terminus, where the interaction between calcium ions and soluble N-ethylmaleimide-sensitive factor activating protein receptor complexes facilitates the docking and fusion of synaptic vesicles containing acetylcholine with the presynaptic membrane, releasing acetylcholine into the synaptic cleft. Acetylcholine binds to densely packed nicotinic AChRs on the cell membrane of the skeletal muscle, opening ligand-gated Na+ channels. This initial flow of Na+ into the cell creates a local depolarization which, if it reaches threshold, activates the opening of voltage-gated Na+ channels. This results in a rapid, large influx of positively charged Na+ ions into cell, changing the membrane potential of the muscle from a resting value between −90 and −70 mV to a contraction threshold of −50 to −40 mV. Membrane depolarization propagates away from the NMJ and reaches T-tubules—invaginations of the cell membrane that dive deep into the body of the muscle—inducing the opening of voltage-gated calcium channels on the sarcoplasmic reticulum, which releases calcium into the muscle. Calcium influx from the sarcoplasmic reticulum facilitates the movement of myosin molecules along actin filaments, shortening the muscle and resulting in contraction. [See Slater, 2015 (6), for an excellent review of NMJ physiology].

## 3 Anatomy

### 3.1 Presynaptic motor neuron terminus

Lower motor neuron axons extend away from the spinal cord, terminating on skeletal muscle as a series of knob-shaped structures, or “boutons” (6). Boutons cluster into groups of 10–100, typically located about midway along the length of the muscle. The interaction of this cluster of boutons with the motor endplate is termed the “motor point” (7). Boutons are divided into regions specialized for synaptic vesicle exocytosis, termed active zones, that alternate with areas specialized for synaptic vesicle endocytosis. Active zones are the site of evoked release of acetylcholine into the synaptic cleft.

### 3.2 Postsynaptic motor endplate

**Figure 1:**
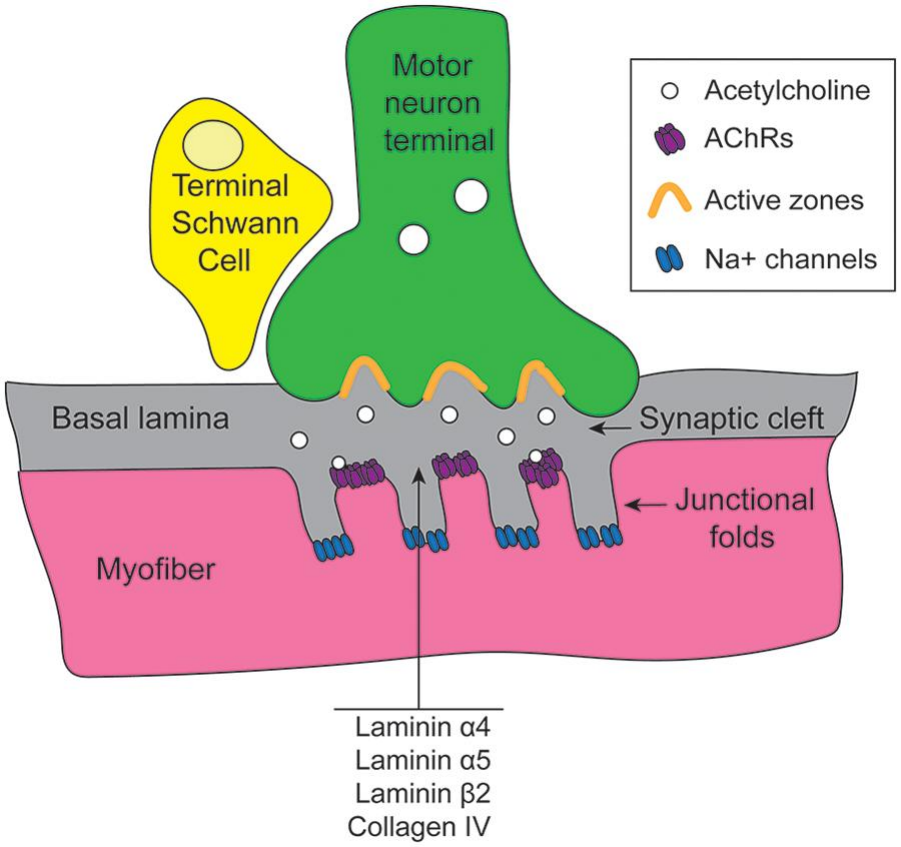
Anatomy of the NMJ. Active zones of the motor neuron terminal align with AChR-rich crests of junctional folds on the motor endplate. Motor neuron terminals release acetylcholine into the synaptic cleft, a specialized basal lamina made up of laminins *α*4, *α*5, and *β*2 and collagen IV. Terminal Schwann cells envelop the NMJ complex. NMJ, neuromuscular junction; AChR, acetylcholine receptor.

The postsynaptic motor endplate is a specialized region of the skeletal muscle cell membrane highly enriched with AChRs. At the motor endplate, the cell membrane invaginates to form a trough called the synaptic gutter (8), the floor of which is formed of invaginations called junctional folds. AChRs are located at the crest and partway down the shoulder of the junctional folds; the troughs of the folds have high concentrations of sodium ion receptors (9). The crests of the junctional folds line up precisely with the active zones of the presynaptic motor neuron terminus, facilitating efficient transmission of acetylcholine (Figure 1).

### 3.3 Synaptic cleft

**Figure 2:**
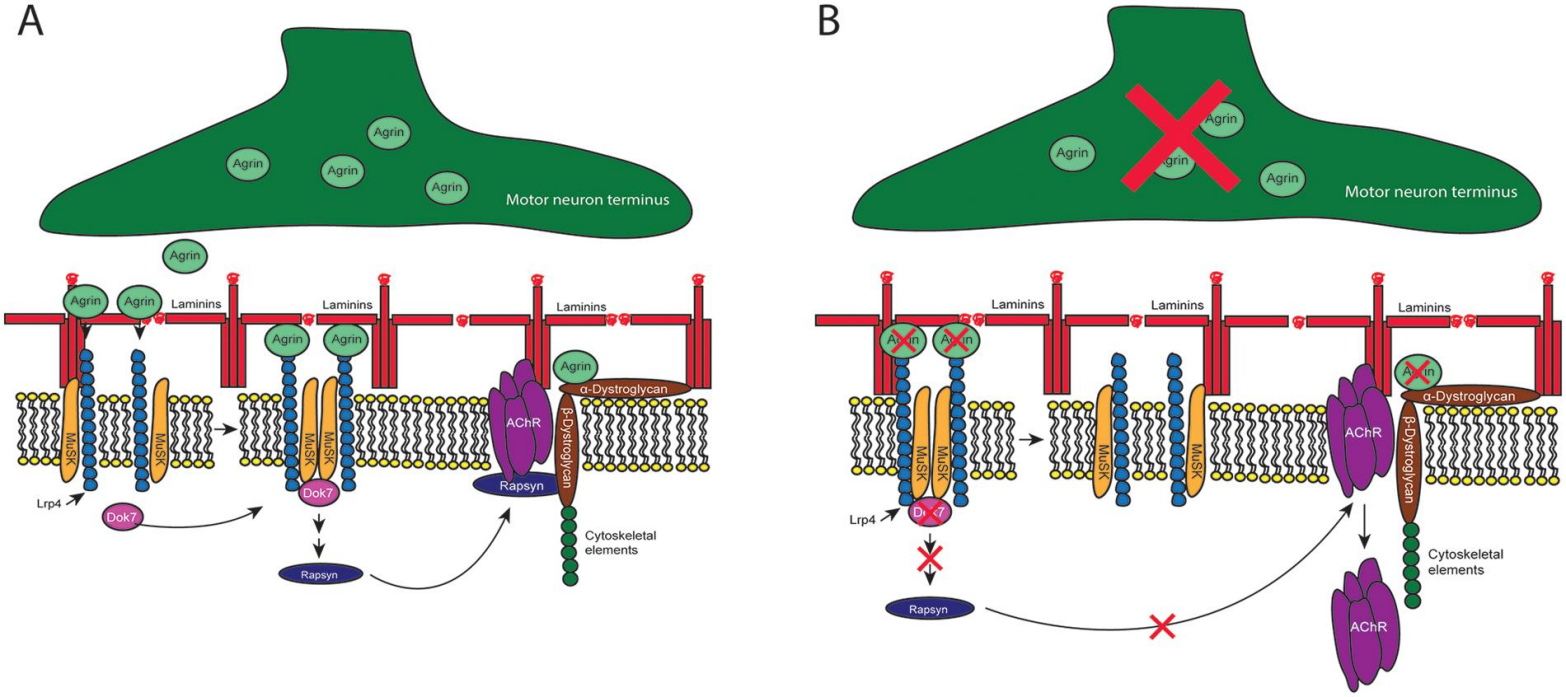
**(A)** Agrin-Lrp4-MuSK-rapsyn complex. Agrin released from the motor neuron terminal binds Lrp4 to form a binary complex that dimerizes to form a tetramer. This results in binding of Dok7 to MuSK, fully activating MuSK and initiating a second messenger cascade that in turn leads to activation of rapsyn. Rapsyn anchors AChRs to the plasma membrane and myofiber cytoskeleton via the dystrophin glycoprotein complex, which also interfaces with laminins. Lrp4, low-density lipoprotein receptor related protein 4; MuSK, muscle-specific kinase; AChR, acetylcholine receptor. **(B)** Following denervation, absence of agrin signaling from the motor neuron results in loss of the Lrp4/agrin tetramer and loss of the Dok7 binding site on MuSK. Rapsyn is not activated, and AChRs become detethered from the plasma membrane.

Skeletal muscle fibers are surrounded by a layer of ECM, termed the basement membrane. The basement membrane is made up of an inner layer, or basal lamina, which directly interacts with the plasma membrane of the muscle fiber and is composed primarily of collagen IV and laminins, and an outer layer, or fibrillar reticular lamina (10). The components of the basal lamina self-assemble and are linked to the cytoskeleton of muscle fibers via integrins and dystroglycan, which bind laminin. The basal lamina also has binding sites for reticular lamina components, creating an interconnected network spanning the cytoskeleton and plasma membrane of the muscle fiber and the outer reticular lamina (10). These linked elements provide tensile strength to muscle fibers (11) and are critical for normal muscle development (12) and maintenance (Figure 2) (13).

The synaptic cleft is a 30- to 50 nm space separating the motor neuron terminus from the motor endplate that is filled in large part by a specialized zone of basement membrane. At the synaptic cleft, only the basal lamina passes between the nerve ending and the motor endplate, extending deep into the junctional folds. Because membrane-associated adhesion molecules are not typically long enough to span the 50 nm gap, the NMJ-associated basal lamina almost certainly plays an important role in maintaining the tight connection between the pre- and postsynaptic portions of the NMJ (10). Experiments in which muscle fibers were digested but the basal lamina was kept intact demonstrated the persistence of motor neuron terminals at sites corresponding with the prior motor endpoint even in the absence of myofibers, underscoring the importance of the basal lamina in aligning the pre- and postsynaptic components of the NMJ (14).

### 3.4 tSCs

Schwann cells (SCs) develop from neural crest cells and comigrate with motor neurons as they travel to skeletal muscle. Myelinating SCs encircle axons in myelin, speeding the conduction of nerve transmission. At the synapse, however, specialized, non-myelinating SCs (i.e., tSCs), envelop the motor nerve terminal, its opposite endplate, and the intervening synaptic cleft.

Ion channels and receptors for neurotransmitters located on SC processes allow SCs to sense and react to activity at the synapse, as evidenced by increases in SC intracellular calcium ion and changes in SC gene expression in response to nerve firing (15). In frog NMJs, blockade of G protein signaling combined with purinergic, peptidergic, and muscarinic tSC receptors minimizes synaptic depression after high-frequency stimulation, allowing for greater synaptic responsiveness, suggesting that tSCs have a modulatory effect on neurotransmission at the NMJ (16). tSCs are also believed to play important roles in NMJ formation, maintenance, and regeneration after injury (17).

## 4 Development

### 4.1 Postsynaptic differentiation

During skeletal muscle development, when myoblasts fuse to form myotubes but before motor neuron innervation, myonuclei begin to express genes encoding the 4 subunits of the embryonic AChR (*α*, *β*, *γ*, and *δ*) (18). In mice, between embryonic day 12.5 and 14.5, two *α* subunits and single *β*, *γ*, and *δ* subunits insert into the muscle cell membrane as pentameric receptors in small clusters near the center of the muscle fiber, a phenomenon termed muscle prepatterning (19–22).

#### 4.1.1 The agrin–low-density lipoprotein receptor-related protein 4 muscle-specific kinase–rapsyn pathway

The arrival of the motor neuron terminal augments early, muscle-directed prepatterning, allowing AChRs to become highly concentrated (10,000–20,000 receptors/μm^2^) directly adjacent to the presynaptic motor axon, leaving them nearly absent (<0 receptors/μm^2^) on the remaining 99.9% of the muscle surface (10, 17). In mice, this clustering of AChRs to the postsynaptic membrane occurs about embryonic day 18.5 (22, 23) and is induced in part by the release of agrin from the motor nerve terminal. Neural agrin is a heparin sulfate proteoglycan synthesized by motor neurons, transported down the axon, and released into the synaptic cleft (24).

The C-terminal domain of agrin binds to low-density lipoprotein receptor-related protein 4 (Lrp4) (25, 26) in the muscle cell membrane, forming a complex that dimerizes to form an agrin-Lrp4 tetramer (27). Formation of the tetramer creates an active site for the binding of Dok7, an adaptor-like protein, on muscle-specific kinase (MuSK), a receptor tyrosine kinase in the muscle cell membrane. The binding of Dok7 and MuSK relieves MuSK autoinhibition, resulting in its full activation, which induces a second messenger cascade that activates rapsyn, a cytoplasmic scaffold protein whose activity is necessary for AChR clustering (28, 29). Rapsyn is thought to anchor AChRs to the muscle fiber cytoskeleton via the dystrophin glycoprotein complex (DGC), which contains a transmembrane dystroglycan complex. Importantly, agrin can bind to the DGC within the synaptic cleft via a binding site for alpha dystroglycan, further stabilizing AChR localization to the postsynaptic membrane (Figure 2) (30).

Before the early 2000s, it was thought that agrin, and thus motor neuron signaling, was necessary for NMJ formation; however, the discovery of prepatterned AChRs in aneural muscles challenged this paradigm (20, 21, 31, 32). In zebrafish, clustering of AChRs on the postsynaptic membrane was found well in advance of motor neuron arrival, even before the motor neuron left the spinal cord (33).

Molding of the postsynaptic membrane by acetylcholine provides a framework by which the function of agrin in NMJ formation might be better understood. Acetylcholine release decreases AChR density in the postsynaptic membrane by altering AChR stability in the cell membrane and decreasing AChR gene expression in muscle fibers through depolarization-induced increases in intracellular calcium concentration. An influx of calcium into the muscle activates calpain, a cysteine protease, which in turn activates cyclin-dependent kinase 5, inducing remodeling of the myofiber cytoskeleton and AChR cluster dispersal (34). Greater levels of intracellular calcium are also thought to activate calcium/calmodulin-activated protein kinase II, which phosphorylates myogenin, a potent transcription factor, blocking its binding to AChR gene promoter regions and decreasing AChR expression (35).

Agrin likely acts in opposition to acetylcholine signaling by stabilizing AChR clusters that have come into contact with motor nerve terminals. Not only does the agrin–Lrp4–MuSK–rapsyn signaling pathway firmly link AChRs to the postsynaptic membrane, but agrin also recruits calpain to rapsyn, inhibiting its activity (36). Thus, agrin signaling from the motor neuron acts as an “anti-declustering” factor rather than (or in addition to) inducing AChR clustering (31).

#### 4.1.2 ECM effects

Components of the ECM also play important roles in sculpting the postsynaptic membrane. Matrix metalloproteinase 3 (MMP3) is a zinc-dependent enzyme housed in the ECM that cleaves agrin, lowering its concentration and thus its anti-declustering activity (37). Importantly, MMP3 is enriched in the ECM surrounding tSCs but is not found in active synaptic zones (37–39). It is possible that MMP3 is secreted or activated by tSCs at the periphery of the synapse, decreasing extrasynaptic agrin and thus concentrating the effect of agrin at the postsynaptic membrane (40).

### 4.2 Presynaptic differentiation

Although postsynaptic differentiation is better understood, it is clear that several elements of the NMJ, including components of the postsynaptic membrane, play important roles in inducing specialization of the presynaptic motor neuron terminal during NMJ formation.

#### 4.2.1 Lrp4

Muscle Lrp4, which binds agrin and MuSK at the postsynaptic membrane, has been implicated in presynaptic differentiation (27). Lrp4 knockout mice display abnormal and impaired motor neuron terminals, active zones, synaptic vesicles, and miniature endplate potential frequency (41, 42). Additionally, cultured cells expressing muscle Lrp4 can induce the formation of small active zones when they come into contact with synaptic vesicles in axons (41, 42). Lrp4 is also important in guiding motor neuron terminals to prepatterned AChR clusters: in Lrp4 mutants, growing motor axons bypass existing AChR clusters, extending into extrasynaptic areas (42).

#### 4.2.2 Beta-catenin and *Wnt* signaling

Beta-catenin is a signaling protein of the canonical *Wnt* signaling pathway. *Wnt* signaling affects NMJ development; however, because different *Wnts* appear to be either positive and negative regulators of NMJ formation, the *Wnt* pathway cannot be described monolithically (40). The effect of beta-catenin in presynaptic differentiation, by contrast, has been more clearly elucidated using mice null for muscle-specific beta-catenin. Motor axon terminals in these mice grew into muscle tendons rather than the center of myofibers, the length and number of secondary nerve branches were decreased, and spontaneous and evoked acetylcholine release from the motor axon terminal was diminished (43). Interestingly, AChR clusters were still present in the middle of the muscle fiber, suggesting that prepatterning of the postsynaptic membrane was still intact and that muscle-specific beta-catenin affects presynaptic differentiation in a retrograde fashion.

#### 4.2.3 Fibroblast growth factors

Fibroblast growth factors (FGFs), including FGF7, FGF10, and FGF22, interact with receptors on the motor axon terminal and promote synaptic vesicle clustering (44). Interestingly, this phenotype is only observed in neo- and perinatal mice, suggesting the importance of FGF signaling for initial NMJ formation but not for postnatal maintenance.

### 4.3 NMJ maturation and stabilization

As embryogenesis progresses, the NMJ undergoes a dramatic transformation. Early in development, clustered AChRs have a relatively uniform, smooth, oval appearance, lacking synaptic gutters or junctional folds. During the late embryonic and early postnatal period, AChRs within the clusters are selectively lost, resulting in multiple “perforations” within the NMJ and the development of a characteristic pretzel-like appearance (45, 46). The loss of AChRs at the motor endplate is concurrent with the arrival of tSCs to the NMJ and synapse elimination, during which all but one axonal input to each NMJ is pruned. Simultaneously, invagination of the floor of the NMJ results in the creation of synaptic gutters and junctional folds (46). As the NMJ continues to mature, active zones are carefully aligned with clefts of the junctional folds, minimizing the distance between synaptic vesicle release from the motor nerve terminal and densely clustered AChRs of the postsynaptic membrane. Finally, the embryonic *γ* AChR subunit is replaced by the adult *ε* subunit.

#### 4.3.1 Synapse elimination

Early in development, NMJs are often polyinnervated, with as many as 10 motor axon terminals converging on a single synapse (47). In mice, during the first two weeks after birth, NMJs undergo a dramatic pruning that weeds the competing motor neuron terminals down to a single neural input (47). This is a dynamic process, with the “losing” motor neuron retreating from the NMJ as a “retraction bulb,” leaving behind synaptic organelles that are engulfed by tSCs (48–50). The complexity underlying this process makes it very difficult to predict which nerve terminal will ultimately “win.” It has been shown, for example, that some motor neurons that have already formed retraction bulbs can grow back to innervate a NMJ if their competitor is eliminated (51).

How tSCs determine which motor axon will innervate a NMJ is likely associated with the ability of the tSC to detect differential neurotransmitter release from the competing motor axon terminals. Darabid et al. (52) demonstrated that a single tSC at a dually innervated NMJ can detect firing from either motor neuron terminal and that intracellular calcium levels within the tSC are proportional to the firing strength of the motor axon.

#### 4.3.2 Conformational changes of the postsynaptic membrane

The changes that lead to the mature, pretzel-like appearance of the postsynaptic membrane are likely closely linked with the mechanisms involved in synaptic elimination. In areas of the postsynaptic membrane where a competing motor nerve terminal has withdrawn, the absence of agrin signaling likely results in destabilization of the AChR cluster, causing endocytosis of AChRs and loss of receptor density.

The role of tSCs in synaptic elimination may also contribute to postsynaptic remodeling. It has been observed that tSCs not only remove “losing” motor axons from the synapse, but that their processes enter the synaptic cleft, preventing axonal interaction with the postsynaptic membrane. This exacerbates the loss of stabilizing agrin signaling, augmenting AChR loss at extrajunctional portions of the synaptic membrane (53).

Conformational changes in the postsynaptic membrane are also driven by *α* laminins within the synaptic basal lamina. Laminins, which, with collagen IV, are the primary components of the basal lamina of the muscle fiber, are heterotrimers comprising *α*, *β*, and *γ* subunits. Laminins specific to the synaptic basal lamina contain *α*4, *α*5, and *β*2 subunits. Laminin *α*5 null mice demonstrate delayed transformation of the embryonic AChR plaque to the mature “pretzel” conformation, whereas laminin *α*4 and laminin *α*5 double mutants demonstrate complete arrest of NMJ maturation (54). These laminins likely work in concert to aggregate dystroglycan to the postsynaptic membrane (54), where it can stabilize AChR within the membrane, potentially through direct binding of agrin and interactions with rapsyn via the DGC (55) (Figure 2).

#### 4.3.3 Development and alignment of presynaptic active zones and postsynaptic junctional folds

It is thought that postsynaptic laminin *β*2 binds presynaptic active zone proteins to bring the postsynaptic membrane into apposition with active zones, facilitating efficient neurotransmission across the synaptic cleft. Fox and colleagues (44) demonstrated that, although laminin *β*2 is not necessary for NMJ formation, it is required for maturation. In mice lacking laminins with *β*2 subunits, motor neuron terminals have only rare active zones, cannot concentrate synaptic vesicles to active zones, and have lower activity at the synapse overall. Additionally, tSC processes in these mutants invade the synaptic cleft, blocking activity (56).

Laminins that contain the *α*4 subunit, which are found primarily at the crests of junctional folds, may play a similar role in orienting the NMJ, because mice that are deficient in laminin *α*4 have normal postsynaptic and presynaptic differentiation but fail to accurately align active zones on the motor neuron terminal with junctional fold crests enriched in AChR on the postsynaptic membrane (23).

Collagen IV, the primary collagen of the synapse, has been shown to help cluster synaptic vesicles in the postnatal motor neuron terminal. Collagen XIII, a membrane-associated collagen, may have roles in both pre- and postsynaptic maturation; mice that are collagen XIII null have aberrant AChR clustering, fewer active zones, and improperly aligned junctional folds and active zones (57).

#### 4.3.4 AChR subunit changes

In mice, approximately 1 week after birth, the *γ* subunit of the AChR is replaced by the adult *ε* subunit, resulting in a shorter period of channel opening but greater calcium ion permeability (58). In rodents, this transition takes place concurrent with the pruning of excess neurons, and no *γ* subunit is expressed into adulthood. In humans, by contrast, the switch to the *ε* subunit takes place approximately week 31 of gestation (59), weeks after motor neuron pruning. Additionally, low-level expression of the *γ* subunit is maintained throughout life (60).

The trigger for this transition is unclear—the *γ* to *ε* switch occurs on its predicted timetable even in denervated muscle (61). It is possible, therefore, that the AChR subunit switch is a process endogenous to the myofiber itself (18).

#### 4.3.5 AChR stability

AChRs cycle in and out of the postsynaptic membrane at a much slower rate in adult NMJs (10-day half-life) than in embryonic clusters (1-day half-life), making the mature NMJ far more metabolically stable than the developing NMJ (62, 63). The various changes occurring during NMJ maturation also lend the NMJ relative resilience to denervation. Although loss of motor neuron innervation triggers rapid disassembly of NMJ plaques during the first postnatal week, AChRs in adult rodents remain clustered for several weeks, even after the onset of muscle atrophy (18).

## 5 Response to peripheral nerve injury

### 5.1 Motor neuron regeneration

Shortly after peripheral nerve injury, axons in the proximal nerve stump degenerate back to the closest node of Ranvier and seal off their cell membrane. After a latent period of approximately 36–48 h, the cytoskeletal structure of the distal stump undergoes rapid, asynchronous fragmentation that proceeds at a rate of 24 mm/hour (64). Axonal breakdown triggers the dedifferentiation of previously myelinating SCs into a phagocytic phenotype to begin clearing degenerated axon and myelin products. SCs and other local non-neural cells also start expressing cytokines and chemokines that attract circulating immune cells, particularly macrophages, to the site of injury for continued removal of cellular debris.

Approximately 2 days after injury, SCs in the distal stump proliferate and begin expressing neurotrophic factors that, elongating parallel, longitudinally align along the empty endoneurial tubes called bands of Bungner, which act as scaffolds to guide regenerating axons into their appropriate downstream fascicular sheaths (65–67). In cases in which the injured nerve is in continuity, axons from the proximal stump follow the bands of Bungner down the distal endoneurial tube, regenerating at a rate of 1 mm/day before arriving at the NMJ (5).

### 5.2 Changes in the postsynaptic membrane

In rodents, 18 days after denervation, AChRs are once again present on extrajunctional regions of the muscle cell membrane. Initially, the number and density of junctional AChRs remain unchanged when compared with controls (68, 69). However, these junctional AChRs are much less stable in the postsynaptic membrane than those at an innervated NMJ, rapidly cycling out of the membrane with a half-life of approximately 1 day (68, 69), mirroring AChR developmental instability. In mice, the loss of junctional folds (70) and the AChR subunit switch from the mature *ε* subunit to the fetal *γ* subunit (71) occur within days of denervation. Approximately 4–5 weeks after the loss of innervation, the rapid removal of AChR receptors from the postsynaptic membrane outpaces cycling of the receptors to the membrane, and the area of the motor endplate begins to shrink.

Fumagalli and colleagues (72) examined the impact of denervation, tetrodotoxin-induced paralysis, and electrical stimulation on rat motor endplate size and stability. Tetrodotoxin only blocks the transmission of electrical signals across the synapse, allowing the passage of trophic factors from the presynaptic to the postsynaptic membrane and limiting the impact of evoked muscle activity on AChRs. The study found that, after denervation or tetrodotoxin treatment, AChR turnover at the postsynaptic membrane increased comparably, suggesting that the lack of an electrical impulse is responsible for AChR instability at the membrane. Supporting this conclusion, rats that underwent denervation followed by direct muscle stimulation had AChR stability of the postsynaptic membrane similar to that of controls. Other studies demonstrated that the loss of junctional folds and the mature AChR subunit after denervation was remedied by direct muscle stimulation (71). Taken together, these data suggest that AChR stability, junctional folding, and mature AChR subunit type are lost after a relatively short period of denervation because evoked muscle activity is lacking, whereas overall AChR number is maintained for a relatively longer period of time as a result of trophic factors from the motor neuron (72).

Neural agrin may be one of these trophic support molecules. After denervation, mice lacking MMP3 (which typically cleaves agrin at the synapse) demonstrate persistent activation of MuSK, preservation of junctional AChR number and size, persistence of the motor endplate at the center of the muscle, and better functional recovery compared with wild-type mice (73).

Signaling from skeletal muscle may also affect remodeling of the motor endplate after denervation. In healthy skeletal muscle, histone deacetylase 4 is found at the plasma membrane, localized to the NMJ. After denervation, however, histone deacetylase 4 relocates to the nucleus, where it relieves suppression of the transcription factor myogenin, leading to increased expression of AChR subunits (74). Suppression of myogenin by acetylcholine is also relieved after denervation, further augmenting AChR subunit expression by myonuclei and explaining, in part, the preponderance of extrajunctional AChRs after loss of innervation (75).

### 5.3 Changes at the synapse and collateral sprouting

After denervation, tSCs begin to divide, adopting a phagocytic phenotype and clearing myelin debris from the synapse. They also extend long cytoplasmic processes through endoneurial tubes, guiding regenerating axons back to their original postsynaptic sites and forming bridges to nearby denervated synapses (76–79). Normally, a single motor axon innervates a single muscle fiber, forming a motor unit. After denervation, however, surviving motor axons send sprouts to neighboring denervated synapses along the tSC-derived bridges in a process known as collateral sprouting (80), expanding their motor unit size roughly fivefold (81–86).

Experimentally, muscle inactivity secondary to presynaptic (via botulinum toxin or tetrodotoxin) or postsynaptic (via bungarotoxin) blockade has been shown to induce collateral sprouting (87). By contrast, direct electrical stimulation of muscle inhibits collateral sprouting, specifically by blocking the formation of tSC bridges (88). It has been hypothesized that muscarinic AChRs present on tSC cell membranes respond to activation by synaptic acetylcholine, inhibiting SC bridge formation at functioning NMJs.

Ultimately, the process of collateral sprouting and the formation of enlarged motor units make up for the loss of approximately 80% of the original motor axons (89). However, when fewer than 20% of the original number of motor axons are available for reinnervation, this compensatory capacity is exceeded, some muscle fibers remain denervated, and weakness becomes apparent.

## 6 Prolonged denervation

On a cellular level, delays in reinnervation result in a loss of AChR stability and density at the postsynaptic membrane, as well as waning of the pro-regenerative state supported by SCs in the distal nerve stump and at the NMJ. In rodent models, by 5 weeks of denervation, tSC-derived cytoplasmic processes begin to retract away from motor endplates (90), limiting collateral sprouting. Growth factor production by SCs in the distal nerve stump also declines such that, by 12 weeks, there is insufficient trophic support for regenerating axons (91, 92). After approximately 6 months of denervation, distal nerve stump SCs atrophy, resulting in loss of their basal laminae and the bands of Bungner necessary to guide regenerating axons toward the NMJ (93).

The ability of SCs within the distal nerve and NMJ to maintain their repair phenotype depends on the expression of the transcription factor c-Jun (94). After nerve injury in rodents, c-Jun expression in SCs continuously increases, peaking around week 3 (95). However, if denervation persists past 5 weeks, c-Jun expression wanes, and SCs become senescent, losing their ability to express pro-regenerative neurotrophic factors. Senescent SCs also take on a senescence-associated secretory phenotype, resulting in the release of pro-inflammatory and ECM-degrading factors that inhibit axonal regeneration (96). Promising work has demonstrated the restoration of SCs to a repair phenotype through neurotrophin 3-induced expression of c-Jun in the SCs of chronically denervated distal stumps, resulting in improvement in axonal regeneration and NMJ reinnervation and decreased muscle atrophy compared with controls (95). Others have demonstrated that the use of systemic senolytic agents to eliminate senescent SCs is associated with enhanced axonal regeneration and better functional outcomes after chronic denervation (97).

In 1944, Gutmann and Young (98) demonstrated that, after prolonged denervation, regenerating axons escape their intramuscular nerve sheaths and begin growing directly into denervated muscle. Building on this work, Fu and Gordon (89) transected the common peroneal nerve in two groups of rats: in the first group, they immediately sewed the nerve directly into the anterior tibialis; in the other group, they waited 6 months and then performed a tibial nerve transfer. They found similarly poor functional outcomes in both groups, despite the availability of fresh axons in the tibial nerve transfer group. In the context of Gutmann and Young's findings, Fu and Gordon suggested that the deterioration of intramuscular nerve sheaths after prolonged denervation allows axons to wander into nearby muscle rather than being guided to the NMJ, precluding motor recovery.

In another set of experiments, Sakuma et al. (99) investigated changes at the rodent NMJ after shorter periods of denervation and found poor functional recovery after only 5 weeks of failed reinnervation. Using high-powered microscopy, they observed the formation of structurally normal NMJs but found that these NMJs could not produce muscle contraction. These findings suggest that, even before the degeneration of intramuscular nerve sheaths, denervation may result in a synaptic failure that limits functional recovery. The mechanisms behind this possible synaptic failure remain to be elucidated.

Denervation is also associated with significant changes in the myofibers of the target muscle, with rapid degeneration of muscle fibers starting as early as a few hours after nerve injury. Molecularly, the loss of motor innervation leads to the activation of FOXO transcription factors, which are typically sequestered in the cytoplasm, resulting in transcription of genes in both the ubiquitin-proteasome and lysosomal/autophagy pathways (100). Expression of Fbxo32 and Trim63, genes encoding striated muscle–specific E3 ubiquitin ligases MAFbx and MuRF1, respectively, results in disassembly of the muscle sarcomere (101). Expression of genes of the autophagy/lysosomal pathways results in degradation of cytoplasmic proteins and organelles, including mitochondria and the sarcoplasmic reticulum.

On a cellular level, denervation induces atrophy of both type 1 (“slow”) and type 2 (“fast”) myofibers. During acute denervation, myofibers shrink in size and shift in fiber type from slow to fast, often taking on an intermediate, hybrid type (100). As denervation persists, myofibers become devascularized as capillaries are lost secondary to endothelial cell death. Inhibition of muscle collagen breakdown after denervation leads to greater deposition of collagen with the endomysium resulting in progressive fibrosis and, ultimately, replacement of myofibers with fat. In rodents, the progression from acute to chronic denervation occurs over the course of 3–6 months, whereas in humans this process takes 6–18 months (100).

In response to muscle atrophy, satellite cells within skeletal muscle are activated, leading to the generation of new myofibers and allowing the muscle to maintain normal numbers of viable muscle fibers. However, as the pool of satellite cells decreases, the ability to compensate for atrophy is increasingly lost and thus, even after delayed reinnervation, muscles often fail to return to their normal size and functionality (89).

It is increasingly clear that the effects of chronic degeneration do not impact the various elements of the NMJ in isolation. For example, Bakooshli et al. (102) recently showed that denervated muscles have increased expression of 15-prostaglandin dehydrogenase, which degrades prostaglandin E2, a molecule essential for satellite cell proliferation. In their study, small molecule blockade of 15-prostaglandin dehydrogenase was associated with increased formation of NMJs and more rapid functional recovery after crush injury in aged mice. Importantly, elevated concentrations of prostaglandin E2 are thought to influence motor neurons in the spinal cord through activation of the cAMP response element-binding protein, suggesting that prostaglandin E2 impacts the survival not only of denervated muscle but also of regenerating axons.

Insulin-like growth factor 1 has also been shown to influence multiple elements of the NMJ after chronic denervation. Hanwright and colleagues (103) demonstrated that sustained delivery of insulin-like growth factor 1 to the distal stump of the transected rat median nerve resulted in less muscle atrophy and greater SC proliferation, likely through mitogenic effects on myocytes and SCs, respectively. Together, these changes were associated with improved functional outcomes after delayed nerve repair.

## 7 Opportunities for investigation

Prolonged denervation is associated with notoriously poor functional outcomes in rodent models and in clinical practice. Despite advances in surgical techniques, Rujis and colleagues (104) found that only half of patients experienced satisfactory motor recovery after surgical nerve repair. The narrow therapeutic window for intervention after peripheral nerve injury has led many to investigate ways to increase the speed of axonal regeneration; however, these efforts have not resulted in substantial improvement in axonal regeneration. Maintaining a stable, healthy NMJ after denervation is an alternate goal that could potentially increase the numbers of patients and the numbers of motor targets for reinnervation after severe peripheral nerve injury.

Avenues for investigation with the goal of maintaining the NMJ after denervation include identifying causes of early synaptic failure and stabilizing the structure of the NMJ, including sustaining the health of intramuscular nerve sheaths and their postsynaptic membranes and countering denervation-associated skeletal muscle atrophy.

### 7.1 Avoiding synaptic failure

In their work, Sakuma et al. (99) found that, early after denervation, axons can successfully regenerate to the NMJ, but the newly reformed NMJ cannot produce muscle contraction. They suggested that this reflects a failure of the NMJ to transition from a regenerative to a synaptic state. This transition is likely highly complex. For example, SCs need to revert from a phagocytotic to a myelinating phenotype to support motor neuron signal transduction, active zones need to realign with junctional folds, and extrajunctional AChRs need to be eliminated. Even the plasma membrane of regenerating axons may need to change upon arrival at the NMJ: by altering the lipid composition, lipid rafts (which house active zones), can become destabilized, a state associated with rapid neurite outgrowth, or become stabilized, which slows axonal regeneration but may facilitate improved active zone functionality (105).

In many ways, successful recapitulation of the NMJ after nerve injury likely mimics NMJ development. Recent work by Mehrotra and colleagues (106) has demonstrated that transient expression of the pluripotency-associated transcription factor NANOG in muscle reprograms myofibers into a more embryonic state. They also demonstrated that expression of NANOG in hindlimb muscles of mice after sciatic nerve transection and repair was associated with increased NMJ formation, decreased muscle atrophy, and improved functional recovery compared with controls. Enhanced expression of genes involved in ECM synthesis and signaling were also seen in NANOG-expressing animals after nerve injury, suggesting that reprogramming of muscle to a more primitive developmental state may help create an environment that is more conducive to synaptogenesis.

Deepening our understanding of how other components of the NMJ, including Lrp4, beta catenin and *Wnt* signaling, and FGFs, function during development and how they respond to nerve injury, may be critical to overcoming early synaptic failure after nerve injury.

### 7.2 Stabilizing the structure of the NMJ

Efforts to stabilize the structure of the NMJ, including AChR clusters and intramuscular nerve sheaths, may play an important role in countering the negative effects of prolonged denervation. MMP3 has been targeted for this purpose because of its role in cleaving agrin at the synapse. A promising study demonstrated that MMP3 knockout animals have prolonged preservation of the motor endplate structure after denervation when compared with controls (73). Understanding the impact of nerve injury on other structural components that anchor the AChR to the muscle cell membrane, including rapsyn, dystroglycans, DGC, and basal lamina laminins, may also yield important insight into how the postsynaptic membrane can be stabilized after denervation.

Maintaining distal stump SCs and tSCs in a repair phenotype, potentially through the induced expression of c-Jun or the elimination of senescent SCs may also help maintain NMJ viability during chronic denervation (95, 97). The senescence-associated secretory phenotype, which is known to have particularly deleterious effects on the ECM, may also contribute to destabilization of the agrin-Lrp4-MuSK-rapsyn complex at the postsynaptic membrane, because it is held in place by laminins within the ECM. Delivery of exogenous SCs to chronically denervated nerve has shown some potential (107–111); however, collection of human SCs requires an invasive nerve biopsy followed by lengthy *in vitro* expansion to produce numbers sufficient for transplantation, which has proved challenging (112). The use of SC-like cells derived from other sources, including skin neural crest–related precursor cells, has shown recent promise in improving recovery after chronic denervation (113).

Electrical stimulation is another possible avenue for investigation. Direct muscle stimulation has been shown to preserve AChR mature subunit type, stabilize AChR turnover at the postsynaptic membrane, and maintain junctional folds. Muscle stimulation also appears to reduce skeletal muscle atrophy in response to denervation, both in rodent models and in the clinical setting (1, 114, 115). The data are mixed, however, with respect to the impact of muscle stimulation on reinnervation, with some groups arguing that electrical stimulation impedes successful reinnervation, resulting in fewer and more polyinnervated motor units (116).

The degree of muscle denervation at the time of stimulation may significantly affect the impact of muscle stimulation on reinnervation. Love and colleagues (88) demonstrated that muscle activity has no effect on the ability of tSCs to extend cytoplasmic processes into empty endoneurial tubules after nerve injury but does impede the ability of tSCs to extend cytoplasmic bridges connecting innervated and denervated synapses. In other words, while a muscle is fully denervated, muscle stimulation should not interfere with tSC outgrowth; however, once reinnervation has commenced, muscle activity impedes collateral sprouting. Supporting this theory, Sinis and colleagues (116) found that prolonged electrical stimulation of paralyzed facial muscles in rats resulted in reinnervation of only one-fifth of the typical number of motor units, a proportion that closely matches the compensatory capabilities of collateral sprouting.

It is also interesting that muscle stimulation is associated with more polyinnervated motor units (116). It is possible that synapse elimination, which is typically guided by tSCs during NMJ development, requires that tSCs assume a developmental phenotype. Muscle activity at the postsynaptic membrane induced by electrical stimulation may cause tSCs to take on a more mature, pro-synaptogenic phenotype, inhibiting this process.

Electrical stimulation of the motor neuron is another avenue for study and intervention. Four randomized controlled trials have demonstrated the efficacy of brief, low-frequency stimulation of the proximal nerve at the time of surgery. Al-Majed and colleagues (117) demonstrated that, after nerve transection and repair, brief electrical stimulation of the proximal nerve stump results in more rapid transit of regenerating axons across the suture line, although not down the distal nerve stump. Wong et al. (118) studied 36 patients with digital nerve transection who underwent epineurial repair followed by implantation of electrodes at the proximal portion of the nerve. In the recovery area, 18 patients underwent a single, one-hour session of electrical stimulation at 20 Hz and had more rapid recovery of temperature discrimination, two-point discrimination, and Semmes-Weinstein monofilament testing compared with 18 patients who underwent sham stimulation. Gordon and colleagues (119) demonstrated that brief postsurgical electrical stimulation of the proximal median nerve after carpal tunnel release was associated with significant increases in motor unit number estimation, less median distal motor latency, and larger median sensory nerve action potential amplitudes compared with controls. Proximal stimulation of the ulnar nerve after cubital tunnel decompression was also shown to significantly increase motor unit number estimation, as well as grip and pinch strength in the stimulated compared with nonstimulated groups (PMID 31432080). Finally, Barber et al. (120) demonstrated that intraoperative stimulation of the spinal accessory nerve during neck dissection for patients with cancer was associated with significantly better subjective and objective scores of shoulder function.

Although less attention has been paid to stimulation of the distal stump, Li et al. (55) demonstrated that distal stump stimulation after sciatic nerve transection results in accelerated Wallerian degeneration, as evidenced by increased degradation and clearance of myelin, more robust recruitment of macrophages and monocytes to the site of injury, and enhanced dedifferentiation of SCs from mature to repair phenotypes. Distal stump electrical stimulation also appears to speed the migration of SCs to the site of injury; functionally, rats who underwent distal stimulation had less atrophy of the gastrocnemius compared with controls. More recently, Lin et al. (121) demonstrated that a single session of electrical stimulation of the distal stump after sciatic nerve transection and repair in rats was associated with improved NMJ preservation, muscle mass, and functional outcomes, potentially because of enhanced activation of muscle satellite cells.

Electrical stimulation of both denervated muscle and nerve has a complex impact on the NMJ and its components, and this impact is likely dependent on a variety of parameters, including timing and stimulation target. Future studies might test whether electrical stimulation can be used not only to maintain the integrity of denervated myofibers but also to selectively induce pro-regenerative vs. pro-synaptogenic phase shifts over the course of nerve recovery. Additional barriers to widespread utilization of electrical stimulation include the need for invasive surgery for the placement of electrodes on injured nerves, which is particularly infeasible in the case of closed, blunt injuries. The development of more refined surface electrodes or placement of ultrasound-guided percutaneous leads may help overcome these challenges. Recent work has also demonstrated that an individual's genetic makeup may impact the efficacy of electrical stimulation on functional recovery. Walters and colleagues (122) demonstrated that rats containing Val66Met single-nucleotide polymorphism within the gene encoding brain-derived neurotrophic factor did not have the same improvement in NMJ reinnervation following electrical stimulation after sciatic nerve transection and repair that was observed in rats without the single-nucleotide polymorphism. Importantly, this single-nucleotide polymorphism is present in nearly 20% of humans globally, underscoring the potential importance of a personalized medicine approach to the management of peripheral nerve injury.

## 8 Conclusion

The inability to reconstitute a functional NMJ after prolonged denervation represents a tremendous impediment to recovery after peripheral nerve injury, limiting not only the time period during which surgical repair can be pursued but also potential targets for reinnervation. Even as surgical techniques and technologies have improved, the speed of axonal regeneration remains limited to approximately 1 mm/day. Efforts aimed at maintaining the integrity of the NMJ beyond 18 months may allow for improvement in functional recovery despite slow axonal outgrowth. In a recent review, Burrell and colleagues (123) identified maintenance of the NMJ and its components as a top priority for study in peripheral nerve injury because of the potential of the NMJ to affect functional recovery, current therapeutic limitations, and opportunities for innovation. A deeper understanding of the NMJ and its response to injury may prove pivotal in improving the prognosis of patients with severe peripheral nerve injury.
